# Marked variation in MSP-1_19 _antibody responses to malaria in western Kenyan highlands

**DOI:** 10.1186/1471-2334-12-50

**Published:** 2012-03-01

**Authors:** Kingsley Badu, Yaw Asare Afrane, John Larbi, Virginia Ann Stewart, John Waitumbi, Evelina Angov, John M Ong'echa, Douglas J Perkins, Guofa Zhou, Andrew Githeko, Guiyun Yan

**Affiliations:** 1 Center for Global Health Research, Kenya Medical Research Institute, Kisumu, Kenya; 2 Department of Theoretical and Applied Biology, College of Sciences, Kwame Nkrumah, University of Science & Technology, Kumasi, Ghana; 3 Walter Reed Project, United States Army Medical Research Unit-Kisumu, Kisumu, Kenya; 4 Division of Malaria Vaccine Development, United States Military Malaria Vaccine Program, Walter Reed Army Institute of Research, Silver Spring, MD 20910, USA; 5 Center for Global Health, University of New Mexico, Albuquerque, NM, USA; 6 Laboratory of Viral and Parasitic Diseases, University of New Mexico/KEMRI, Centre for Global Health Research, Kisumu, Kenya; 7 Program in Public Health, College of Health Sciences, University of California at Irvine, Irvine, CA 92697, USA

## Abstract

**Background:**

Assessment of malaria endemicity at different altitudes and transmission intensities, in the era of dwindling vector densities in the highlands, will provide valuable information for malaria control and surveillance. Measurement of serum anti-malarial antibodies is a useful marker of malaria exposure that indicates long-term transmission potential. We studied the serologic evidence of malaria endemicity at two highland sites along a transmission intensity cline. An improved understanding of the micro-geographic variation in malaria exposure in the highland ecosystems will be relevant in planning effective malaria control.

**Methods:**

Total IgG levels to *Plasmodium falciparum *MSP-1_19 _were measured in an age-stratified cohort (< 5, 5-14 and ≥ 15 years) in 795 participants from an uphill and valley bottom residents during low and high malaria transmission seasons. Antibody prevalence and level was compared between different localities. Regression analysis was performed to examine the association between antibody prevalence and parasite prevalence. Age-specific MSP-1_19 _seroprevalence data was fitted to a simple reversible catalytic model to investigate the relationship between parasite exposure and age.

**Results:**

Higher MSP-1_19 _seroprevalence and density were observed in the valley residents than in the uphill dwellers. Adults (> 15 years) recorded high and stable immune response in spite of changing seasons. Lower responses were observed in children (≤ 15 years), which, fluctuated with changing seasons particularly in the valley residents. In the uphill population, annual seroconversion rate (SCR) was 8.3% and reversion rate was 3.0%, with seroprevalence reaching a plateau of 73.3% by age of 20. Contrary, in the valley bottom population, the annual SCR was 35.8% and the annual seroreversion rate was 3.5%, and seroprevalence in the population had reached 91.2% by age 10.

**Conclusion:**

The study reveals the micro-geographic variation in malaria endemicity in the highland eco-system; this validates the usefulness of sero-epidemiological tools in assessing malaria endemicity in the era of decreasing sensitivity of conventional tools.

## Background

Malaria still thrives in the African highlands, in spite of low vector density exposure [1]. The western Kenya highlands are an area of particular interest based on the fact that on a relatively small spatial scale, there is considerable variation in altitude, water accumulation, and land-use patterns. As a consequence, the epidemiology of malaria varies markedly. For example, small differences in altitude have been noted to lead to large differences in suitability and availability of vector breeding habitats, and consequently, differing risks of malaria transmission and prevalence [2,3]. These patterns of malaria reflect heterogeneities in vector distribution, human vector-contact, and human host factors [4]. Identified risk factors for malaria transmission include distance to known mosquito breeding sites [5,6], household construction methods [7], and personal protection measures against mosquito bites [8]. Moreover, altitude and environmental landscape, i.e., topography have also been correlated with risk of malaria infection [2,4,9-11].

Assessing variation in malaria endemicty at different altitudes across regions with differing malaria transmission intensities can be achieved directly by determining exposure to malaria-infected mosquitoes, the entomological inoculation rate (EIR) [12], or indirectly by evaluating serological evidence of malaria exposure in the human population [13,14]. Direct measure of the EIR becomes difficult when absolute numbers of mosquitoes and sporozoite rates are low, particularly when EIR is below the detection limits of commonly used trapping methods [15,16]. The situation is further complicated when the mosquito densities show marked heterogeneity, because spatial and temporal variations in mosquito densities necessitates long-term intensive and extensive sampling to be accurate [15-17]. Direct determination of malaria parasite prevalence in the human population as an indicator of malaria transmission intensity has limited sensitivity when transmission is low [18-20], furthermore, the sensitivity of the tools used in routine detection of parasitemia; microscopy and *Pf*HRP2 based rapid diagnostic test (RDTs) presents additional challenges at low parasite densities.

Prevalence of antibodies to *Plasmodium falciparum *has been explored as a marker of human exposure to malaria [13,14,21-24]. Measurement of serum antibodies is a useful index of malaria transmission intensity when the focus is on evaluation of malaria exposure over time, since anti-malarial antibodies develop after repeated exposures and can persist for months to years after infection [14]. Seroprevalence reflects cumulative exposure and thus it is less affected by seasonality or unstable transmission due to the longer duration of the specific antibody response. Additionally the longevity of antibody response generates a seroprevalence that is higher than equivalent parasite rates, making it a more sensitive measure. Therefore, immunological markers may be useful to detect malaria exposure in areas of low endemicity [21,24]. Seroconversion rates are related to the force of infection of malaria as refracted through the immune responses of exposed individuals [24-26]. Thus the seroconversion rates provide measures of malaria exposure that compares with the malaria transmission intensity [13,14,27]. Additionally, antibody responses have been shown to have a tight correlation with EIR and offer the potential to detect recent changes in malaria transmission intensity [13,14,27].

However, the use of inadequate serological markers may underestimate exposure by virtue of their lack of sensitivity. For instance, in the case of circumsporozoite protein (CSP), sporozoites injected by infected mosquitoes have a relatively short life-span in the blood. Some rapidly develop into liver stages and others are taken up by macrophages, processed, and presented to the immune system [23]. The amount of antigenic material and the time of contact with immuno-competent cells are relatively shorter than blood stage antigens and thus may underestimate malaria exposure in low transmission settings. Druilhe and others could not detect CSP antigens in children in low transmission area even though 78% of them had detectable blood stage antigens, and from these data, they concluded that CSP is not a reliable marker of malaria endemicity when the total EIR in the area is less than 10 infectious bites per person per year [28]. In hyperendemic areas, however, CSP has been reported to give reliable estimates of malaria endemicity and reflects the seasonal dynamics of transmission [23,29] and may be sufficiently sensitive to evaluate the protective efficacy of anti-vector devices in transiently exposed travelers to endemic countries [30]. On the other hand, surface proteins of merozoites like apical merozoite antigen-1 (AMA1), are highly immunogenic and tend to saturate detectable antibody responses in the population in low to moderate transmission settings. Alternatively, Drakeley and others [14] described MSP -1_19 _as the most suitable immunological marker for assessing malaria endemicity at varying altitudes along transmission intensity cline.

MSP-1 is secreted as a 195-kDa precursor anchored via glycosylphosphatidylinositol [31]. This is cleaved by proteases into fragments of 83, 28-30, 38-45 and 42 kDa [32]. During merozoite invasion, the 42 kDa fragment (MSP-1_42_) is further cleaved to produce a 33-kDa fragment (MSP-1_33_) and a 19 kDa C-terminal fragment (MSP-1_19_); with the later remaining attached to the merozoite surface and present on ring forms in newly invaded erythrocytes [33]. MSP-1_19 _is thus a recognized target of protective immunity [34].

This study was therefore conducted to investigate serologic evidence of malaria exposure at a highland site along a malaria transmission intensity cline to characterize differences in malaria endemicity. The current study reports an improved understanding of the micro-geographic variation on malaria endemicity in the complex highland eco-system; and potentially identifies vulnerable groups in the event of hyper-transmission. This study confirms that sero-epidemiology provides valuable information for planning effective malaria control strategies and surveillance systems.

## Methods

### Study site

The study areas included two highland sites located in Iguhu and Lidambiza villages (0°10'N, 34° 44'E, elevation 1,420-1,500 m above sea level [asl], defined as valley bottom site), and Sigalagala and Museno villages (0°33'N, 34° 47'E, elevation 1,520-1,600 m [asl], defined as uphill site), all in Kakamega county, western Kenya. The terrain of the study area is typical of the highlands and consists of hills and river valleys. The hillside is mostly dotted with maize fields and subsistence crops. Six different seasonal streams flow within the valleys in the study villages and empty into the main Yala River, which runs through the area from east to west. The inhabitants live in houses of stick frames with mud walls and thatch or corrugated metal roofs. The area experiences two rainy seasons, and averages 2,000 mm rainfall per year. The long rainy season typically occurs between April and May, with an average monthly rainfall of 150-260 mm, while the short rainy season typically occurs between September and October, with an average monthly rainfall of 165 mm. The main dry season occurs from December to March [35]. The mean annual daily temperature is 20.8°C. Malaria prevalence peaks usually 1-2 months after the onset of the rains. Malaria vectors in the area are *Anopheles gambiae *sensu stricto and *A. funestus*, [4,36]. Most mosquito larval habitats are found on riverbanks in the bottom of the valley and on the banks of streams during both dry and rainy seasons. Adult mosquito population thus tends to cluster around the valley bottom, where about 90% have been found within a distance of 300 meters of the breeding sites at the valley bottom [4,35,37].

### Study participant and blood collection

A serological cross-sectional survey was carried out during the dry season in February-March 2009, and again in the rainy season in June-July of the same year, corresponding to the low and high transmission seasons, respectively. To obtain informed consent, residents in individual, randomly selected households were approached, the study goals and procedures were explained and potential participants were invited to enroll in the study. All willing individuals signed consent forms or provided a thumbprint in the presence of witnesses. Parental consent was obtained for children and all participants were transported to the Iguhu district government hospital. Individuals of all age groups from the study sites were eligible to participate except infants of less than 6 months of age. Demographic data including age and gender were taken, and, as altitude was a critical feature of the study, participants were also required to confirm their village of residence. Subsequently, clinicians and nurses obtained venipuncture blood (~ 3 ml). Sera was isolated and transported to the Kenya Medical Research Institute (KEMRI) Kisumu laboratories for storage in freezers at -80°C until further processed. Thick and thin blood smears were prepared according to standard protocols described elsewhere [38], to determine parasite prevalence. All individuals presenting with fever and parasitemia were given free malaria treatment by a clinician according to Kenya government guidelines, and children under the age of five were also given free ITNs as per Ministry of Health policy. The study was approved by the Kenya Medical Research Institute Ethical Review Committee [SCC No. 1382(N)] and the Institutional Review Board of the University of California, Irvine.

### Measurement of specific humoral responses

The expression and purification of the *Pf*MSP-1_19 _FVO recombinant protein has been described elsewhere [39,40]. Total IgG responses to *Pf*MSP-1_19 _FVO specific antigen were measured in serum by indirect ELISA [27]. Briefly, plates were coated with 0.2 μg antigen in 100 μl of 1xPBS, and incubated overnight at 4°C. After blocking with 0.5% casein, 0.05% Tween 20, test sera were serially diluted in triplicate on the plates from 1:50 to 1:64,000 and incubated for 2 h at 22°C, followed by horseradish peroxidase-conjugated goat anti-human IgG (KPL, Gaithersburg, MD) for 1 h. After the addition of ABTS peroxidase substrate (Kirkegaard & Perry Laboratories Inc., Gaithersburg, MD), plates were incubated for 1 h, at 22°C and the reaction stopped by addition of 10 μl of 20% SDS (Sigma, St. Louis, MO). Plates were washed 4 times between each step. Plates were read at 414 nm with the *SPECTRAMAX *340pc (Molecular Devices Corporation, Sunnyvale, USA) and the serial dilutions were used to fit a four-parameter curve using SoftMax Pro v4.1 (Molecular Devices). Results were expressed in titer values, the titer endpoint being defined in this study as the calculated serum dilution yielding an optical density of 1.0. A serum pool obtained from 30 naïve US donors never exposed to malaria was used to define an assay cutoff; the mean titer +3 SD represented the cutoff value for all negative responses to the *Pf*MSP-1_19 _antigen. The resultant titer value obtained as cutoff was 63.3; any titer above this value was considered as positive. A pool of malaria positive individuals from hyper-endemic areas in Kenya was used for positive controls.

### Statistical analysis

MSP-1_19 _seroprevalence is the number of positive responders out of the total number of participants tested; this is simply referred to in the whole text as seroprevalence. Differences in the proportion of seroprevalence of MSP-1_19 _between age-stratified, uphill and valley residents were compared by the χ^2 ^test with *p *< 0.05 considered statistically significant. The Mann-Whitney test was used to test if medians of seroprevalence were different between localities. Multinomial logistic regression was used to examine the association between MSP-1_19 _seroprevalence and parasite prevalence adjusting for age in the valley and uphill population. Linear regression was used to examine the trend of parasite prevalence and age at different localities

We fitted age specific MSP-1_19 _seroprevalence data to a simple reversible catalytic model using the maximum-likelihood method that assumes a binomial error distribution;

$$P_{t}=\frac{\lambda }{\lambda +\rho }\left(1-e^{-\left(\lambda +\rho \right)}\right)$$

where *P_t _*is the proportion of individuals aged *t *that is seropositive, constant λ is the annual rate of seroconversion and ρ is the annual rate of reversion to seronegative. This was done to investigate the relationship between force of parasite exposure and age [14]. For *Pf*MSP-1_19 _antibody titer, the data were analyzed and graphed using GraphPad Prism software (San Diego, CA, USA). The age-seroprevalence model was fitted using JMP 9.0 (SAS, Cary, NC 27513, USA).

## Results

### Study population

The distribution of the participant's population in terms of age and locality are shown in Table 1

**Table 1:** MSP-1_19 _seroprevalence (%) in different age groups stratified according to season and locality in western Kenya Highland

Uphill						Valley				
**Season**	**< 5** **(n = 88)**	**5-14** **(n = 174)**	**≥ 15** **(n = 143)**	χ^2^-value**	***P***	**< 5** **(n = 83)**	**5-14** **(n = 151)**	**≥ 15** **(n = 160)**	χ^2^-value**	***P***

Dry	27.50	36.70	75.00	30.34	< 0.001	60.00	70.00	98.40	45.59	< 0.001

Rainy	25.00	49.10	64.80	17.21	< 0.001	71.20	87.10	90.70	11.56	0.003

χ^2^-value*	0.067	2.402	1.770			1.007	6.496	3.686		

*P*	0.794	0.120	0.180			0.315	0.010	0.054		

* Comparison between seasons, df = 1 for all tests

** Comparison among different age groups, df = 2 for all tests

### Parasite prevalence

**Figure 1:**
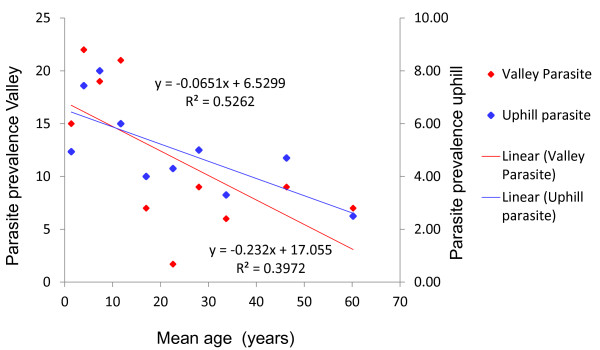
**Scatter graph showing trends of parasite prevalence with age in the uphill and valley populations**.

In Figure 1, generally, age-parasite prevalence trends correlated negatively with each other. In the uphill residents, significant age-related parasite prevalence was observed where parasite prevalence decreased with increasing age (R^2 ^= 0.52, p = 0.02). A similar trend was observed in the valley bottom residents where parasite prevalence generally declined with increasing age, however, this was marginally significant (R^2 ^= 0.40, *p *= 0.05; Figure 1). There were marked differences in parasite prevalence between uphill and valley residents. These differences were significant in the < 5 and 5-14 years age groups, (*χ*^2 = ^3.93, df = 1, p = 0.047) and (*χ*^2 ^= 9.26, df = 1, *p *= 0.002) respectively. In these two groups we observed parasite prevalence as high as a 2-fold increase in the valley residents compared with the uphill residents. However in the adult group we observed no significant differences in parasite prevalence between uphill and valley residents (*χ*^2 ^= 1.93, df = 1, *p *= 0.164).

### Variation of MSP-1_19 _seroprevalence with age, season, and altitude

In Table 1 when the two sites were considered independently, there was significant variation in seroprevalence with age for both sites during both seasons; proportions of seropositive individuals increased significantly with increasing age. Seasonal variation was generally observed at both localities; however, it was only in the valley bottom area where significant seasonal variation in seroprevalence was observed in the 5-14 years age group.

Furthermore, highly significant differences were found when seroprevalence was compared between the uphill and valley residents in both seasons (dry season χ^2 ^= 33.78, df = 1, *p *< 0.001, rainy season χ^2 ^= 69.96, df = 1, *p *< 0.001). However, when the two sites were combined there was no significant inter-seasonal variation in seroprevalence in < 5 and ≥ 15 years age groups (*p *= 0. 170 and *p *= 0.190, respectively). In contrast, the seasonal variation in seroprevalence was significant in children within the 5-14 years age group (χ^2 ^= 10.73, df = 1, *p *= 0.001).

### Spatiotemporal variation in total IgG titers

**Figure 2:**
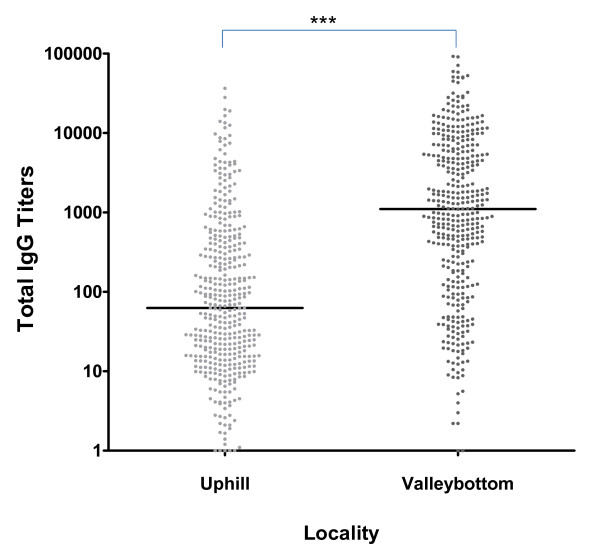
**Differences in IgG titers among different localities**. *** indicates the significance test by Mann-Whitney test, *P *value < 0.001 (Uphill n = 401, Valley n = 394).

Analyses of median IgG titer levels among the study population revealed a13-fold higher titer levels in residents at the valley bottom compared to the uphill population (Mann-Whitney test, z = 13.17, *P *< 0.0001), (Figure 2). Generally, increasing MSP-1_19 _seroprevalence correlated positively with increasing relative antibody levels; in the Uphill population (R^2 ^= 0.847, *p *< 0.001,) and in the valley population (R^2 ^= 0.623, *p *= 0.011). Adjusting for age, the multinomial logistic regression used to assess the association between MSP-1_19 _seroprevalence and parasite prevalence, revealed that seropositive individuals were more likely to have been exposed to parasites compared to seronegative individuals (Table 2). This was true, both in the uphill and valley residents.

**Table 2:** Showing the association between MSP-1_19 _seroprevalence with *Plasmodium *parasite prevalence at the different localities

	Parasite prevalence		
**MSP-1_19 _seroprevalence**			

***Locality (n)***	***Odds Ratio***	***95% CI***	***P value***

Uphill (401)	2.798	[1.018, 7.693]	0.046

Valley (394)	3.167	[1.196, 8.386]	0.020

Total Uphill and Valley (795)	4.282	[2.200, 8.330]	< 0.001

### Age-dependent antibody acquisition model

**Figure 3:**
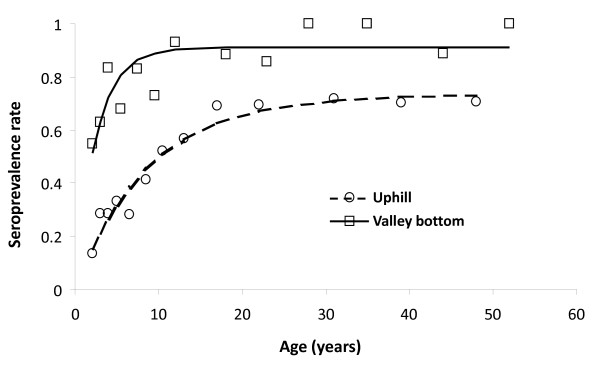
**Kinetics of the age-dependent antibody prevalence at different altitudes**. phill model: *P*_t _= 0.73(1-e^-0.11t^), *R*^2 ^= 0.95, *P *< 0.001; valley bottom model: *P*_t _= 0.91(1-e^-0.39t^), *R*^2 ^= 0.67, *P *< 0.001.

A simple catalytic model showed that seroconversion and seroreversion rates were highly age-dependent (Figure 3). In the uphill population, the annual seroconversion rate was 8.3% and reversion rate was 3.0%, with seroprevalence reaching a plateau of 73.3% by the age of 20 years (probably due to less frequent exposure to parasite infection). In contrast, the valley bottom population annual seroconversion rate was 35.8% and annual seroreversion rate was 3.5%, and by 10 years of age 91.2% of the population already had antimalarial antibodies (Figure 3).

## Discussion

Using age-specific MSP-1_19 _seroprevalence, seroconversion rates (SCR), and total IgG titers, together with malaria infection prevalence, we have observed considerable variation in human exposure to malaria in western Kenya. Seroprevalence of residents at the valley bottom was almost two fold higher than that of the uphill residents. Comparison of the median total IgG titers in the study population revealed a13-fold difference between the uphill and valley bottom residents. Similar trends have been observed in entomological and parasitological studies, which have consistently found higher transmission intensity, vector abundance, and parasite prevalence at the valley bottom, in comparison to the top of the hill [4,17,38]. This may partly be explained by the differences in water accumulation between the two sites, as the valley bottom residents live along River Yala which provides permanent breeding habitats for malaria vectors all year round, resulting in a higher risk of exposure to infected mosquitoes compared to residents uphill [4,10,17,38].

Immunity to malaria is a function of exposure challenge; it develops gradually as a consequence of experiencing multiple parasite exposures or persistent infection for years. Consequently, seroprevalence reflects cumulative exposure and, thus, is less affected by seasonality or unstable transmission due to the longer duration of the specific antibody response [14,21,41]. It may be limited in detecting discrete seasonal variation in transmission but is a good indicator of long term transmission potential. And antibody persistence generates seroprevalence that are higher than equivalent parasite rates making it more sensitive [14,27]. Thus seroprevalence at this site particularly in the valley residents indicate possible frequent or persistent exposure to parasite infection. An earlier study at this same site reported that, 38.2% of asymptomatic individuals harbor infections that persist for 2-5 months and a further 14.2% of them harbored asymptomatic infections from 6-12 months [17]. A follow up study revealed high infection turn-over rate i.e. frequent clearance and acquisition of infection, with the average infection duration of single parasite genotypes being 1.1 months, and the longest genotype persistence being 3 months [10]. Whether these infections are recrudescence or re-infections they have the ability to maintain seropositivity and thus lead to acquisition and maintenance of significant high levels of antimalarial antibody responses. Moreover, it has been observed that persistent or even sub-patent infections are sufficient to maintain seropositivity and partial immunity consistent with the concept of premunition [42].

Age-specific seroprevalence has been used to estimate seroconversion rates (SCR) as a measure of malaria transmission intensity. Earlier studies in neighboring Tanzania have shown that these estimates are tightly correlated with EIR measurement [13,14,27]. Age sero -prevalence curves reflect different levels of transmission intensity. In low transmission settings development of antibodies is slow and is mainly exhibited by the adult population, whereas in a high transmission area, much of the population will be seropositive even at a younger age [43]. This phenomenon is clearly demonstrated in our age seroprevalence curves (Figure 3). In the uphill population the seroprevalence reaches a peak of 73.3% only at age 20; this is in sharp contrast to that seen in the valley, where seroprevalence reaches a peak of 91.2% by the age of 10. These observations reveal the difference in the intensity of malaria transmission between the two localities, suggesting a higher intensity of malaria transmission in the valley area than the uphill area. These findings are further corroborated by other evidence such as the vector density variation between valley bottom and uphill in our study site [4,17] and by the repeated infections observed in the valley area [10,17].

In very low transmission settings, where parasite prevalence and EIR are insensitive, serological measures offer a way of accurately assessing endemicity and identifying focal areas of transmission supporting the potential for elimination [43]. Historically, several studies have used serology in this context. In determining endemicity and evaluating eradication campaigns in Tanzania, repeated cross-sectional serological surveys were used to assess approximately 1,500 individuals, and the serological data accurately distinguished between areas of different transmission intensity [44]. Similarly in Surinam, supporting an elimination program, approximately 2,000 individuals of all ages were serologically assessed; and the antibody responses in these individuals reflected the epidemiological situation at the time of sample collection and accurately defined areas which had eliminated malaria [45]. Furthermore, in Mauritius and Tunisia, immuno-florescent antibody assays (IFAT) were used to follow reducing seroprevalence over several years in eradication campaigns until no seropositives were detected in children under 5 and 15 years, respectively, thus confirming successful eradication [46,47].

When we compared age-specific IgG titers from the two sites, there was a highly significant difference in the IgG titers between the valley bottom and the uphill residents, indicating a considerable variation in malaria endemicity within the highland area. The observed seasonal variation in the IgG titres in the 5-14 year group suggests that the level of exposure may not result in the development of stable humoral responses by the age 14. If this is solely due to seasonal exposure to malaria parasites, then it may represent a vulnerable group in the valley bottom area that may pose a public health problem in an event of hyper malaria transmission. As individuals remain seropositive for several years, the level of antibody response (IgG titers) can reflect fluctuations in recent exposure. It is known [43] that antibody levels tend to be higher in actively infected individuals with a concomitant decline as the parasites are cleared. This seems to be the case in the less than 15 years age group in the valley who also had highest parasite prevalence. The valley bottom is characterized by persistent infections, a single parasite genotype has been observed to persist for 3 months [10], detailed examination of IgG titer with age revealed that on the average, titer level at age 5 in valley is similar to that at age 20 in uphill (data not shown). This should not be surprising as at the age of 10 seroprevalence in the valley exceeds 90%.

The observed parasite prevalence in the current study is lower compared to earlier studies conducted in the same area [17,40]. Munyekenye and others [43], reported a mean annual parasite prevalence of 47.0% in children of 1-9 years and 9.5% in > 19 year olds. Baliraine and others [17] using the combination of microscopy and PCR technique observed parasite prevalence of 34.4%, 34.1% and 9.1% in 5-9, 10-14 and > 15 years, respectively. The current conducted two cross-sectional surveys across all age groups of participants, and observed a mean infection prevalence of 14.0% in < 5 and 5-14 year olds and 6.8% in > 15 years. Consistent with all these studies is the finding that parasite prevalence generally decreases with age and distance from the valley bottom. Parasite prevalence in adults has not exceeded 10% for several years, probably due to their ability to clear and suppress parasites through their acquired immunity or from clearance using antimalarials. On the whole the mean parasite prevalence in the valley population and that of uphill population were 16.3% and 6.3% respectively. However the equivalent seroprevalence were 79.56% and 46.34% for valley and uphill respectively. This implies that looking at parasite prevalence alone about 84% and 94% of valley and uphill residents would be considered unexposed. Seroepidemiology thus presents a more sensitive tool in describing the malaria endemicity of a population under low to moderate transmission.

We observed a spectrum of MSP-1_19 _responses that are highly varied within the same age group and altitude transects. Other studies have observed similar variation in exposure, susceptibility and even disease patterns at the individual level [11,48,49]. This finding may partly be explained by factors such as host genetic polymorphism, [50], MSP-1 polymorphism [51] or antigenic sin. Nevertheless, in a population that is predominantly of one sub-tribe, variation in exposure to mosquito bites is likely to play an important role [7]. Intrinsically individual factors such as household structure, use of ITN [8], and proximity to breeding sites [15,22] may be important determinants of this variation. A follow-up study is underway to test this hypothesis by testing individual responses to *An. gambiae *salivary gland protein (gSG6-P1) previously validated as a marker of mosquito bite exposure [52].

## Conclusions

MSP-1_19_, a leading vaccine candidate was used as a marker of immune response and a proxy of parasite exposure between the valley bottom and uphill residents. We observed higher immune response to the *Plasmodium falciparum *antigen in the valley residents than in the uphill dwellers indicating a possible higher exposure to infections. Odds ratios indicated seropositive individuals were more likely to be parasite positive than seronegatives. High and stable humoral immune responses were observed in older residents (> 15 years) at our highland site in spite of changing seasons. However, in children (≤ 15 years) we observed lower responses that also fluctuated with changing seasons. If the MSP-1_19 _immune responses reflect functional immunity, something that was not directly determined in this study and is currently uncertain, uphill populations, particularly children ≤ 15 years of age, may be at a higher risk of (severe) clinical disease. This hypothesis requires further longitudinal studies.

## Competing interests

The authors declare that they have no competing interests.

## Authors' contributions

KB carried out the field survey, the serological experiments, and the serological analysis and wrote the first draft of the manuscript. GY, YA and AG developed the study protocol, YA led the field operations, data and laboratory specimen collection, and GZ supported field team operations and data analysis. JW, JL, EA and AS coordinated laboratory procedures and serological testing, EA provided the plate antigen and participated in the study design and implementation, GY, AG, DJP and JMO conceived the study, participated in its design and implementation and supervised fieldwork, laboratory investigations, data analysis and manuscript development. All authors read and approved the final manuscript.

## Pre-publication history

The pre-publication history for this paper can be accessed here:

http://www.biomedcentral.com/1471-2334/12/50/prepub
